# Impact of Chemical Aging on Venison Processing Knife Topography and Recoverable Chronic Wasting Disease Prion Seeding Activity

**DOI:** 10.3390/pathogens15060645

**Published:** 2026-06-17

**Authors:** Damani N. Bryant, Nicole A. Lurndahl, Maddy Ellis-Cramer, Sarah C. Gresch, Marc D. Schwabenlander, Peter A. Larsen, Tiffany M. Wolf, Stuart S. Lichtenberg

**Affiliations:** 1 Minnesota Center for Prion Research and Outreach, St. Paul, MN 55108, USA; dnbryant@umn.edu (D.N.B.); plarsen@umn.edu (P.A.L.); wolfx305@umn.edu (T.M.W.); 2 Department of Veterinary and Biomedical Sciences, College of Veterinary Medicine, University of Minnesota, St. Paul, MN 55108, USA; 3 Department of Veterinary Population Medicine, College of Veterinary Medicine, University of Minnesota, St. Paul, MN 55108, USA

**Keywords:** prion decontamination, scanning electron microscopy, energy-dispersive X-ray spectroscopy, RT-QuIC, bleach, Briotech, Virkon S, Wex-Cide 128

## Abstract

Infectious prion adsorption on metal, minerals, wood, and plastic is well documented, raising the specter of food safety hazards for meat packing workers, sport hunters, and consumers. We previously demonstrated that sodium hypochlorite, and to a lesser extent, potassium peroxymonosulfate, and hypochlorous acid can decontaminate prion-contaminated nonporous surfaces. However, the extent to which chemical aging of surfaces affects subsequent recoverable prion seeding activity is unknown. In this study, we investigated the potential for four chemical decontaminants known for their anti-prion activity (sodium hypochlorite [bleach], hypochlorous acid [Briotech], potassium peroxymonosulfate [Virkon-S], and Wex-Cide-128) to alter the surfaces of steel knives and the subsequent prion decontamination efficacy of each. We found that hypochlorous acid, sodium hypochlorite, and potassium peroxymonosulfate corrode the surfaces of steel knives, resulting in significant physical alterations. Knives exposed to hypochlorous acid exhibited the most substantial corrosion (rust), which is consistent with its oxidizing effects. Oxidation of the knife surface was corroborated by complementary energy-dispersive X-ray spectroscopy data trends. Scanning electron microscopy data indicate corrosion is apparent after minimal exposure to oxidizing agents. Finally, we used the real-time quaking-induced conversion assay on swabs collected from chemically aged knife surfaces to evaluate recoverable surface-associated CWD-prion seeding activity detected by RT-QuIC after prion exposure and decontamination. Our results indicate decreased recoverable prion seeding activity from knife surfaces aged with 40% bleach. We also observed some recoverable seeding activity post-decontamination on knives chemically aged with 10% bleach and Wex-Cide-128, but largely similar efficacy to prior studies. This implies that existing chemical prion decontaminants are likely effective after repeated use on steel surfaces.

## 1. Introduction

Prion diseases, also known as transmissible spongiform encephalopathies [1], are a class of insidious, progressive, and invariably fatal neurodegenerative diseases triggered by the conversion of the normal, alpha-helical dominant cellular prion protein (PrP^C^) into the misfolded, beta sheet-enriched prion protein (PrP^RES^ or PrP^SC^) [2,3]. A wide range of mammalian species are susceptible to prion diseases, including cattle, cervids, humans and sheep. Prion disease in cervids (deer, elk, moose, etc.), termed chronic wasting disease (CWD), is transmitted vertically and horizontally, with the first evidence of horizontal transmission likely described by Williams and Young in 1982 [4]. Prions shed into the environment are known to persist for more than a decade, likely contributing to complex environmental transmission networks [5]. The combination of direct and indirect exposure routes, along with prion shedding, resistance to protein degradation, and the insidious nature of disease progression, makes CWD one of the most challenging wildlife diseases to manage. This observation is supported by surveillance data, with the United States Geological Survey (USGS) reporting that CWD has been detected in cervids in 36 states and five Canadian provinces as of summer 2025 [6].

Classically, infectious prions accumulate in the nervous system and lymphoid system; however, CWD-afflicted cervids, particularly *Odocoileus* sp., exhibit widespread dissemination of infectious prions in peripheral tissues [7], including muscle [8]. This has profound food safety implications for venison processing. To date, there are no known instances of CWD zoonosis in humans; however, a nonzero risk remains. This concern is bolstered by the precedent of bovine spongiform encephalopathy (BSE, or mad cow disease), which has infected humans since the 1990s and has led to at least 230 cases of the variant Creutzfeldt–Jakob disease [9]. In the classic toxicological framework, risk is the product of hazard and exposure. Although the hazard to human health from CWD appears low currently, exposure rates to potentially infectious prions remain uncomfortably high. Six million white-tailed deer are hunter-harvested annually in the United States alone [10]. The risk of a CWD crossover event is directly associated with hunters’, meat processors’, and consumers’ exposure to prion-contaminated meat, byproducts, and associated processing surfaces [11,12,13,14]. Finally, multiple CWD strains circulating with unknown species boundaries [15] could potentially increase the hazard at some unspecified future date.

Surface swab testing via the real-time quaking-induced conversion (RT-QuIC) represents a viable and timely approach for evaluating prion contamination during cervid processing and decontamination [13,16]. Prion decontamination on nonporous surfaces using sodium hypochlorite (bleach), potassium peroxymonosulfate (Virkon S), hypochlorous acid (Briotech), and Wex-Cide 128 has been documented [17,18,19,20]. In our previous work, we demonstrated that prion contamination of metal and plastic surfaces can cross-contaminate CWD-negative tissues [16]. Further, we found that sodium hypochlorite, and to a lesser extent, potassium peroxymonosulfate and hypochlorous acid can decontaminate prion-contaminated nonporous surfaces. However, the extent to which chemical aging affects subsequent recoverable CWD prion seeding activity is unknown. Many decontaminants, such as bleach and Briotech, are oxidizers and thus are known to corrode metal surfaces, increasing the complexity of surface topography. In light of the data that prion decontamination is less effective on highly porous surfaces [13], it stands to reason that chemical aging might facilitate prion adsorption and decrease decontamination efficacy. Furthermore, chemical changes in steel surfaces may also alter prion affinity [21]. In this study, we evaluated the impact of chemical aging, a byproduct of repeated decontamination cycles, on steel knife surface topography, elemental composition, and recoverable surface-associated CWD-prion seeding activity detected by RT-QuIC.

## 2. Materials and Methods

### 2.1. Prion-Positive Samples

This work was approved by the University of Minnesota Institutional Biosafety Committee (IBC #2512-43558H). A CWD prion-positive venison sample was obtained opportunistically via hunter harvest samples from Wisconsin. Animal care and use protocols were not required by the University of Minnesota Institutional Animal Care and Use Committee under these circumstances. Prion seeding activity in previously frozen prion-positive venison (#59045) was confirmed via Nano-QuIC (Supplemental Figure (Figure S1)) prior to the onset of experiments using the protocol of Li et al. [8]. All CWD-positive tissues and surfaces were handled in HEPA-filtered Class II Type A2 biosafety cabinets in a biosafety level two laboratory at the University of Minnesota. CWD-positive waste material was disposed of via appropriate streams (prion-rated biohazard waste or digester). The same CWD-positive sample was used to contaminate each knife surface in this experiment. Sample #59045 underwent a freeze–thaw cycle for each decontaminant condition over the course of weeks. We did not observe a robust correlation between freeze–thaw cycle and overall seeding activity. Decontaminants were tested in the following order: Briotech, Virkon S, 40% bleach, 10% bleach, and Wex-Cide (see Table 1).

### 2.2. Knife Blade Chemical Aging

**Figure 1:**
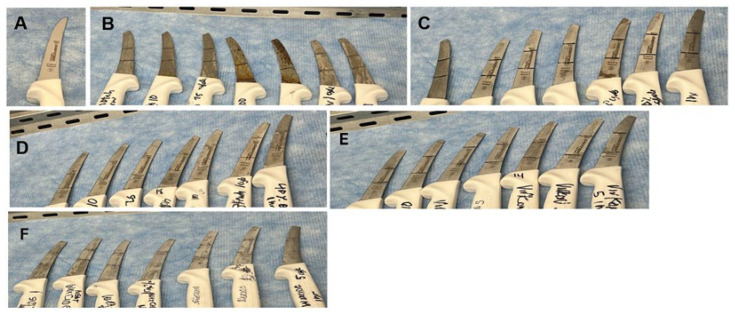
Chemical aging affects the appearance of boning knives, particularly those exposed to the oxidizing agent Briotech. Each boning knife was cycled through one decontaminant for a specific number of times (1, 10, 25, 100, or 500). Another set of knives was cycled 1 or 5 times with 1 h exposure in one decontaminant each. Knives are arranged in the aforementioned order in each image: (**A**) untreated, (**B**) Briotech, (**C**) 10% bleach, (**D**) 40% bleach, (**E**) Virkon S, and (**F**) Wex-Cide.

Commercially available boning knife blades (herein: knife/knives; Dexter-Russell, Southbridge, MA, USA) 01473; proprietary DexSteel blade, high-carbon, and high alloy stainless steel) were chemically aged via repeated exposure to the following decontaminants (Figure 1): bleach (7.5% sodium hypochlorite solution diluted to 10% *v*/*v*/7500 parts per million (ppm) or 40% *v*/*v*/30,000 ppm; https://www.clorox.com/, Oakland, CA, USA); Briotech (0.02% hypochlorous acid solution, neat, https://briotechusa.shop, Waukegan, IL, USA); Virkon S (2% potassium peroxymonosulfate, neat, Lanxess AG, https://lanxess.com, Pittsburgh, PA, USA); and Wex-Cide 128 (o-Phenylphenol and chlorophene active ingredients, neat, https://www.wexfordlabs.com/, Kirkwood, MO, USA). One knife was exposed to each experimental (decontaminant × number of cycles) condition. Tinkerkit Braccio robots (Arduino T050000, Monza, Italy) were fitted with Arduino Uno microcontrollers (Arduino A000066, Monza, Italy) and programmed to move knives through the following protocol: dip in 1 L of decontaminant, air/drip-dry, dip in 1 L of tap water (rinse), and air/drip-dry, for five minutes each. Each knife cycled through one decontaminant for a specific number of times (1, 10, 25, 100, or 500). A separate set of knives was cycled 1 or 5 times through 1 h exposures to one decontaminant each, using the same Tinkerkit Braccio setup (Monza, Italy). The other steps of the cycles were unchanged.

### 2.3. Scanning Electron Microscopy

**Figure 2:**
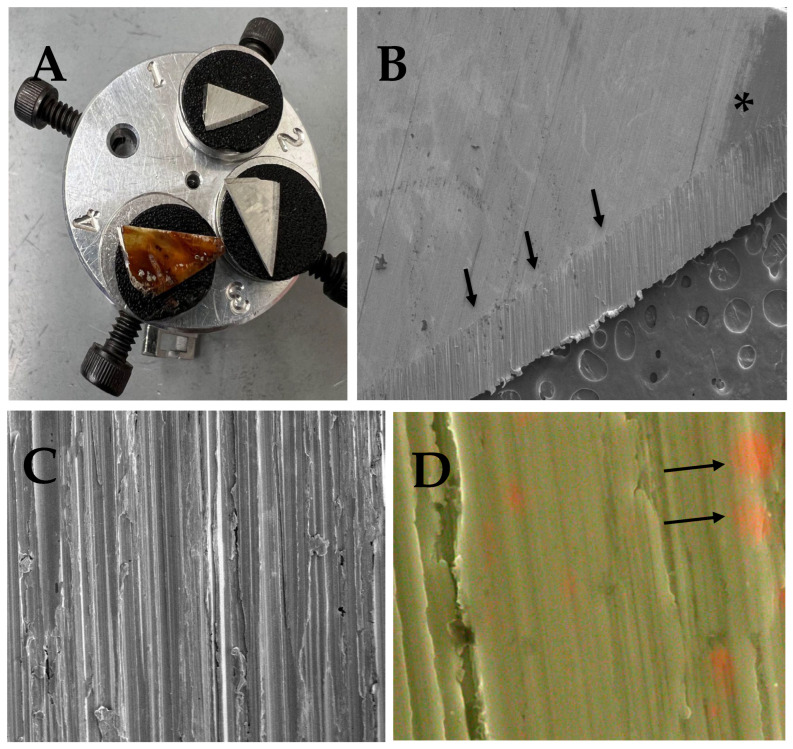
Knife machining, discolorations, and elemental composition are observed in untreated and disinfectant-treated knives using scanning electron microscopy. (**A**) Knife tips were mounted on SEM stages using black-carbon-based adhesive tape for examination using a Hitachi SU8230 Cold Field Emission Gun Scanning Electron Microscope. Clockwise: No treatment, Virkon S (500 cycles), and Briotech (500 cycles). (**B**) Discoloration (asterisk) is observed on the untreated knife blade at low magnification (30×). Knife machining is easily observed on the edge of the knife blade (arrows). (**C**) Higher magnification (350×) image of an untreated knife, showing the machining of the knife. (**D**) EDS elemental map of an untreated control knife that consists of Iron (Fe; %wt: 81.4, green), Chromium (Cr; %wt: 13.72, red, arrows), carbon (C; %wt: 3.4), Molybdenum (Mo; %wt: 0.91), silicon (Si; %wt: 0.49), and Aluminum (Al; %wt: 0.08).

The tip of each knife was removed using a wire cutter (Fastenal St. Paul, MN, USA) and mounted on an electron microscope stage using carbon adhesive tape (Figure 2A) for examination in a Hitachi SU8230 Cold Field Emission Gun Scanning Electron Microscope (Chioda, Tokyo, Japan) at the University of Minnesota Twin Cities College of Science and Engineering Characterization Facility. Electron micrographs were captured using an electron beam that ranged from 1 to 15 kilovolts (kV). The emission current (Ie) was set to 10 microamps (µA) for generating electron micrographs. Magnifications ranged from X30 to X1.5K (Figure 2B,C).

### 2.4. Energy-Dispersive X-Ray Spectroscopy

To evaluate elemental composition in chemically aged knives, energy-dispersive X-ray spectroscopy (EDS) using an Oxford X-Max^N^ 80 with a large area silicon drift detector (Oxford Instruments, High WyCombe, UK) was employed. EDS measures characteristic X-rays generated when an electron is ejected from an atom in response to the incident electron beam. An electron from a higher shell falls into the ejected electron’s shell, resulting in the generation of a characteristic X-ray that corresponds to the energy difference between the two shells. AZtec One software (Version 6.2, Oxford Instruments, High WyCombe, UK) was used for EDS elemental mapping (Figure 2D) under the following conditions: 15kV, 20 µA, frame count =1024, dwell time = 500, dead time ~20%, and a working distance of 15 mm. The relative percentage of each element, by weight (wt%), is also calculated using Aztec One software. EDS wt data (in triplicate) was visualized as a function of the number of decontaminant cycles using GraphPad Prism version 10.4.1 (graphpad.com). Fractional (less than 1%) wt% data were not included in the data analysis.

### 2.5. Knife Surface Prion Contamination Sampling

Chemically aged knives were rinsed with tap water prior to evaluating recoverable prion seeding activity. Using our previously described protocol [16], Swabs (Fisherband PurSwab Foam Swab, Thermofisher Scientific, Waltham, MA, USA) were moistened with 1× phosphate-buffered saline (PBS) before swabbing surfaces and stored at −20 degrees Celsius (°C) in 1.5 mL microcentrifuge tubes afterwards. Knife blades were divided into thirds using a Sharpie and a total of 9 (3 each per 3 conditions) swabs were collected from each knife blade. Knives were swabbed prior to contamination. Knives were subsequently contaminated using the same CWD prion-positive venison sample (#59045). The partially thawed venison tissue was smaller than each surface space. It was smeared generously across each knife using forceps before the knife was swabbed with a new swab. After the contamination swabs were collected, the same venison sample was generously smeared across the knife a second time. The knife was allowed to dry for 30 min at room temperature prior to a 5 min decontamination soak using the same decontaminant used for chemical aging and a 5 min soak in tap water. A third set of swabs post-decontamination was subsequently collected and frozen at −20 °C.

### 2.6. RT-QuIC of Swabs

One swab from one (of three) regions of each knife was tested via RT-QuIC. Four technical replicates were evaluated for each swab. As there was no effect of chemical aging (Figure S2), the data was collapsed across the decontamination cycle (and was not replicated) in order to have sufficient statistical power to evaluate the effect of disinfectant on seeding activity. Swabs were processed for RT-QuIC using a slight modification of previously published work [13,16]. Prions were extracted from swabs as follows. Three hundred microliters (µL) of 0.6 mM myristyl sulfobetaine (MSB; Sigma-Aldrich, Burlington, MA, USA) [22,23,24] were added to each microfuge tube containing a swab. Samples were vortexed, sonicated (3 × 5 s cycles with 5 s rest intervals; QSonica, Newton, CT, USA), and centrifuged at 4 °C for 3 min at 2400× *g* to pellet out debris. Supernatants were centrifuged at 21,100× *g* for 30 min at the same temperature. The supernatant was discarded, and the pellet was frozen at −20 °C or processed for RT-QuIC.

RT-QuIC assays were performed as described in [25]. To create “neat” or 10^0^ samples, pellets were resuspended in 50 µL of RT-QuIC sample dilution buffer, which consisted of a 1× N2 supplement (Fisher Scientific Hampton, NH, USA) and 0.1% SDS in 1× PBS. In total, 5 µL of each 10^0^ sample was further diluted in 45 µL of the RT-QuIC sample dilution buffer to create a 10^−1^ sample. Consistent seeding activity was only observed at 10^0^, so 10^−1^ samples were not analyzed further (Figure S3). In total, 2 ul of the sample was added to 98 μL of RT-QuIC reaction buffer, which consisted of 1× PBS, 500 µM EDTA, 50 µM Thioflavin T (ThT), 300 mM NaCl, and 0.1 mg/mL of the recombinant hamster prion protein (wild type HaPrP90-231) substrate (MNPROtein; Minnesota Center for Prion Research and Outreach, St. Paul, MN, USA). Four technical replicates were tested for each swab, positive plate control and negative plate controls. RT-QuIC assays were run using sealed black 96-well, clear-bottom plates (Thermo Scientific) on a FluoStar Omega plate reader (software version 5.70 R2, BMG Labtech, Cary, NC, USA). Samples were incubated at 42 °C for 48 h, with 60 s of double orbital shaking followed by 60 s of rest. A 448-10 excitation filter and a 482-10 emission filter were used to measure ThT fluorescence every 45 min, with a fixed gain of 1000. Fluorescence was scaled in arbitrary relative fluorescence units (RFUs). The mean RFU of cycles 2 and 3 was used to calculate background fluorescence for each well.

### 2.7. RT-QuIC Data Analysis

MARS data analysis software (version 4.2, BMG Labtech) was used to calculate the rate of amyloid formation (RAF, a measure of prion seeding activity), which evaluates the onset of the exponential phase of each RT-QuIC reaction (16). RAF is the inverse of the time to threshold (TTT; the time in seconds required for RFU to cross the threshold of twice the background fluorescence). Data was subsequently visualized using GraphPad Prism version 10.4.1 (Boston, MA, USA). Since the swab RAF data was not normally distributed, a nonparametric Kruskal–Wallis statistic, followed by Dunn’s Multiple Comparison (Mean Rank Difference) post hoc test, was calculated using GraphPad Prism.

## 3. Results

### 3.1. Effect of Chemical Aging on Knife Surface Topography

Compared to an untreated knife, all knives showed visible discoloration after exposure to each decontaminant (Figure 1).

Briotech had the most pronounced visual effect on knife surfaces, with corrosion/rust proportional to the number of exposure cycles (Figure 1B and Figure 2A). The remaining knives evidenced generally comparable discoloration in response to bleach, Virkon S, and Wex-Cide (Figure 1C–F).

**Figure 3:**
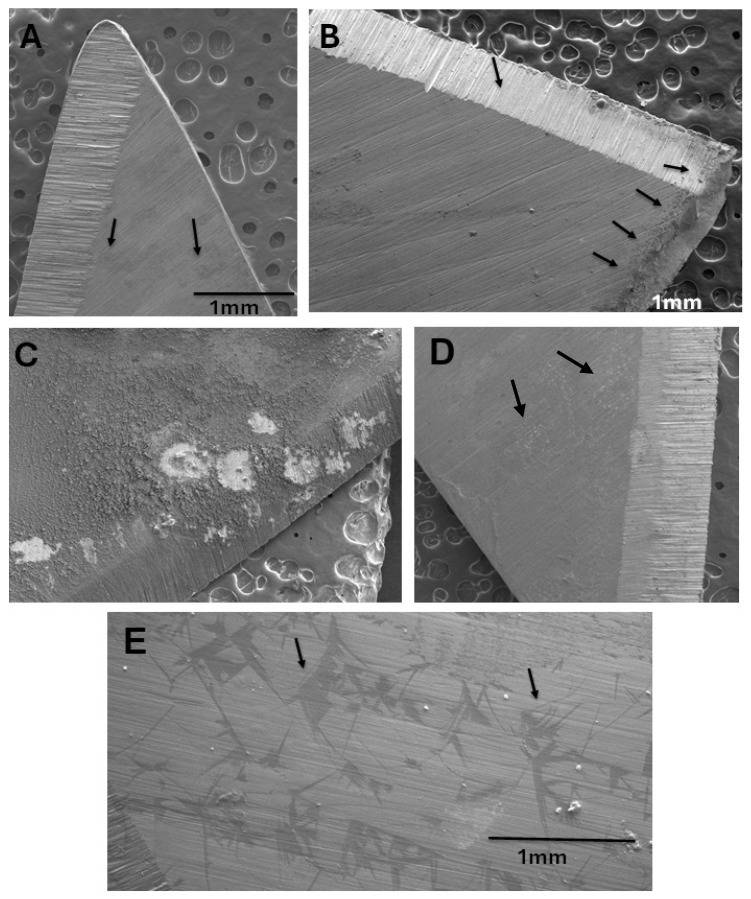
Although all decontaminants affected the knife surface topography, Briotech caused the most extensive changes. (**A**) 10% bleach, 500 cycles, was associated with electron-dense discolorations (arrows). (**B**) 40% bleach, 500 cycles, caused some corrosion (arrows) on the edge of the knife. (**C**) Briotech, 500 cycles, caused the most extensive surface corrosion/rust. (**D**) Virkon S, 500 cycles of exposure, caused surface changes that included electron-transparent regions (arrows). (**E**) Wex-Cide, 500 cycles of exposure, caused electron-dense regions (arrows). All images were captured at 1.0 kV, 30× magnification and a working distance between 10.6 and 13.1 mm.

Not surprisingly, the most extensive changes to knife surface topography at the SEM level were caused by 500 cycles of Briotech (Figure 3C). Electron-dense discolorations were observed with 500 cycles of 10% bleach (Figure 3A). More severe topographical changes were observed on the knife edge with 500 cycles of 40% bleach (Figure 3B). Topographical changes (sometimes electron dense) were also observed on knives exposed to Virkon S and Wex-Cide (Figure 3D,E).

### 3.2. EDS Analysis of Knife Elemental Composition

**Figure 4:**
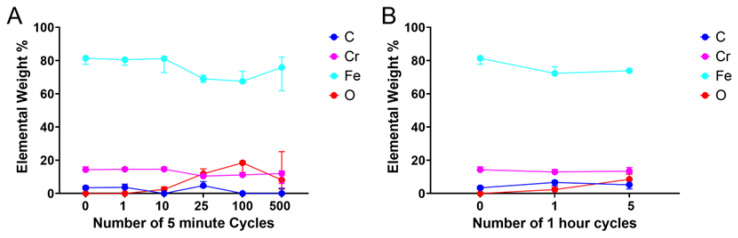
The oxidizing agent Briotech increased the oxygen weight percentage in chemically aged knives. Energy-dispersive X-ray spectroscopy (EDS) analysis of knife surfaces. The elemental weight percentage for all elements above 1% is visualized in triplicate for each knife. The same, untreated control is included in both graphs for comparison (0 number of cycles). (**A**) Increased oxygen (O) was observed in knives exposed to 25 and 100 cycles of 5 min Briotech exposure. A nonparametric statistical analysis indicated that this effect was not statistically significant (Kruskal–Wallis statistic = 0.1255, *p* = 0.9997). (**B**) The same trend is observed in knives exposed to five cycles of 1 h exposure to Briotech.

Elemental weight percentage data (wt%) indicated that, generally speaking, there were no significant changes to the element composition of knives exposed to decontaminants. Iron (Fe), Chromium (Cr), and carbon were typically the most abundant elements, accounting for approximately 80%, >20% and >10% of the elemental weight percentage, respectively, as would be expected of stainless steel [26]. The notable exception was Briotech-exposed knives (Figure 4), which demonstrated an increased oxygen wt%, proportional to up to 100 cycles of exposure. Nonparametric statistical analysis of the data indicated that the trend was not statistically significant relative to the untreated control 0 (Figure 4).

### 3.3. Effect of Chemical Aging on Recoverable CWD-Prion Seeding Activity

RT-QuIC data is summarized in Table 1. We did not observe any effect of decontaminant residue on RT-QuIC data (Figures S3 and S4) as chemically aged knives were rinsed with tap water prior to evaluating surface-recoverable prion seeding activity. Although we hypothesized that there may be a robust effect of “decontamination cycle” on recoverable seeding activity, we did not observe any (Table 1, Figure S2). For our initial semi-quantitative evaluation, we established a threshold: 2/4 or 50% of the technical replicates (TR) needed to demonstrate prion seeding activity for the sample to be designated prion-positive (Table 1). Although RT-QuIC data is known to be stochastic, seeding activity was unambiguous (Figure S4) in this study. The median and range of the rate of amyloid formation data is visualized in Figure S2. Except for 40% bleach, and to a lesser degree, Virkon S, prion seeding activity (contamination) on chemically aged knives was consistent (4/4 TR) across decontaminants. Reduced (2/4 or 50%) seeding activity was observed in knives chemically aged with 40% bleach for 10, 25, and 100 cycles (Table 1, Figure S2B). In most cases, a single treatment with each decontaminant abolished recoverable seeding activity from knife surfaces. There were no instances of prion decontamination failure with 40% bleach or Briotech (Figure S2B,C). However, there were two instances of prion decontamination failure (recoverable seeding in 2/4 TR) with Virkon S (Figure S2D) and one instance of decontamination failure in Wex-Cide (Figure S2A,E).

**Figure 5:**
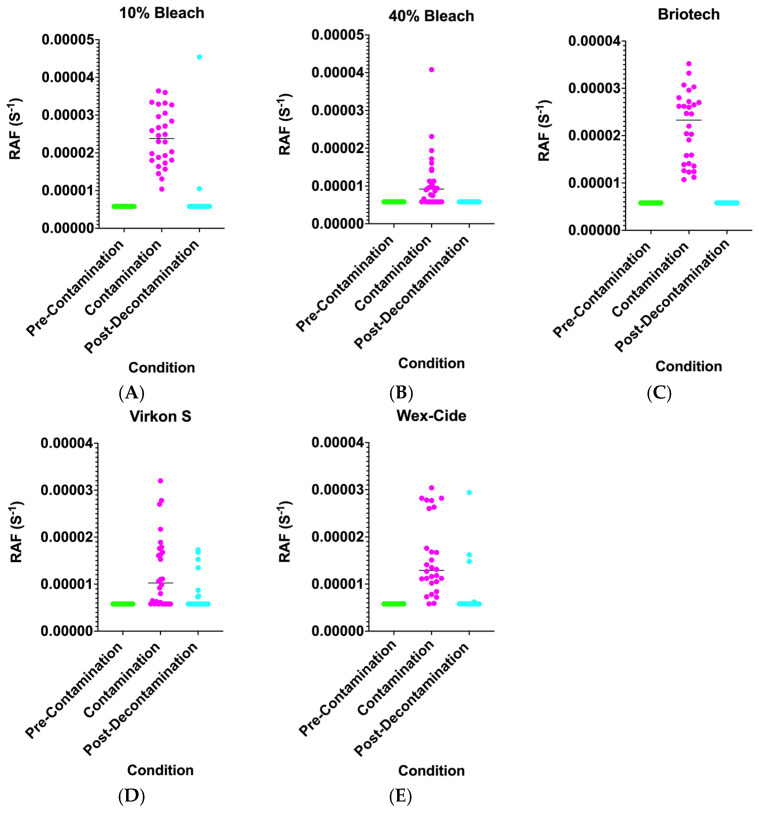
Prion seeding activity varies as a function of the decontaminant used for chemical aging. Chemically aged knives were swabbed before contamination (green), after CWD prion contamination (magenta), and after 35 min of decontamination and a rinse with tap water (blue). All technical replicates are visualized, and the horizontal line indicates the median of the data. (**A**) 10% bleach, (**B**) 40% bleach, (**C**) Briotech, (**D**) Virkon S, and (**E**) Wex-Cide. Statistical analysis of this data is presented in Table 2. Visualization of this data as a function of decontamination cycle is presented in Figure S1.

Data (Figure S2) were subsequently collapsed across “cycle” to statistically evaluate decontamination efficacy (Figure 5). To statistically evaluate CWD-prion-recoverable seeding activity in non-Gaussian data, a nonparametric statistical analysis was performed across the decontaminant conditions (pre-contamination, contamination, and post-decontamination; Table 2). Of all the decontaminants, only Virkon S failed to show a statistically significant difference between “contamination” and “post-decontamination” (Dunn’s Multiple Comparison Test: Mean Rank Difference = 7.000; *p* = 0.679). All other decontamination failures did not reach statistical significance using this test.

## 4. Discussion

In our previous publication [16], we questioned the impact of repeated cycles of disinfection on stainless steel surface integrity and prion adsorption and decontamination efficacy. Evidence from cryogenic electron microscopy experiments suggests that CWD prion fibril surfaces are heterogeneous with respect to solvent-facing residues, with abundant patches of both cationic and anionic moieties [27]. This, in many ways, accounts for the avid interactions between prion fibrils and a host of surfaces with disparate properties. Thus, alterations to the chemical structure of any given surface could radically alter prion adsorption. Although our current data do not provide unambiguous support for this mechanistic thesis, we demonstrate that, with a few notable exceptions, recoverable CWD-prion seeding activity is not correlated with decontaminant-triggered changes to surface topography and chemical composition. It is important to note that with the data presented here, we cannot distinguish prion adsorption on surfaces from recovery. Further, though our results are consistent with prior studies, we note that these experiments are exploratory in nature, and further confirmation and validation of this work is warranted prior to adoption of specific decontamination protocols. Repeated exposure to Briotech produced the most corrosive changes to the knife surface topography. Elemental oxygen levels trended upward in knives treated with Briotech, consistent with its oxidizing activities. However, prion seeding activity and decontamination were not affected by Briotech chemical aging or decontamination, respectively. Although 40% bleach was less corrosive than Briotech, prion contamination was less consistent on knives aged with this decontaminant. Brief (5 min) exposure to Briotech or bleach (at sufficient concentration) is sufficient to effectively and reliably reduce prion seeding activity on knives (and other impermeable surfaces) used during venison processing. Ten percent bleach, Virkon S, and Wex-Cide were less effective than the other chemicals in decontaminating knives under these conditions. It is important to reiterate that the detection of prions on chemically aged knives in this study only reflects recoverable CWD-prion seeding activity, but not infectivity. It is quite possible that intact and biologically active prions remain on steel surfaces following decontamination using our described protocols. For these reasons, bioassays using the appropriate animal model are required to evaluate prion infectivity.

There are two parallel and commingled issues that occur in the context of venison processing: CWD testing of hunter-harvested deer prior to consumption, and biosafety issues associated with processing animals of unknown CWD status. With respect to the first issue, USDA-validated diagnostic tests, such as the enzyme-linked immunoassay (ELISA) and immunohistochemistry (IHC), exist for CWD surveillance. These validated tests are also recommended for making consumption decisions when venison is harvested from CWD-positive cervid populations [28,29]. Furthermore, results from rapid, more sensitive assays such as RT-QuIC are potentially available within 48 h, allowing venison consumers to make educated decisions until the ELISA/IHC assays are complete. In the future, RT-QuIC could streamline harvest testing prior to submission of deer to processors, thereby enabling the establishment and enforcement of guidelines around the disease status of harvests that are accepted into the processing facility. With respect to food biosafety, the acceptance of deer of unknown CWD status into processing facilities is a potential source of significant cross-contamination. In the absence of prior testing, decontamination is the most reliable tool for preventing prion cross-contamination of venison, processing implements, and processing facilities until CWD status is ascertained.

Taken together, the current findings extend our understanding by providing evidence that chemical aging does not enhance recoverable CWD-prion seeding activity despite altering the knife surface topography. This study reinforces effective decontamination options that may be viable for such facilities. Our data continue to undergird the decision-making process for establishing best practices for the handling and processing of post-harvest venison. However, RT-QuIC negativity should not be interpreted as proof of complete loss of infectivity, and bioassays are required to evaluate residual infectious risk. We emphasize this point in light of the biosafety implications of our work and strongly encourage future work to address bona fide infectivity using animal models.

## Figures and Tables

**Table 1:** Summary of RT-QuIC data. Ratio of CWD prion-positive technical replicates to total technical replicates tested. Seeding in 2/4 or greater technical replicates is the threshold for designating the sample as prion-positive (red).

	**10% Bleach**		
	**Pre-Contamination**	**Contamination**	**Post-Decontamination**
1 Cycle	0/4	4/4	0/4
10 Cycles	0/4	4/4	1/4
25 Cycles	0/4	4/4	1/4
100 Cycles	0/4	4/4	0/4
500 Cycles	0/4	4/4	0/4
1 Cycle 1 h	0/4	4/4	0/4
5 Cycles 1 h each	0/4	4/4	0/4
	**40% Bleach**		
	**Pre-Contamination**	**Contamination**	**Post-Decontamination**
1 Cycle	0/4	4/4	0/4
10 Cycles	0/4	2/4	0/4
25 Cycles	0/4	2/4	0/4
100 Cycles	0/4	0/4	0/4
500 Cycles	0/4	4/4	0/4
1 Cycle 1 h	0/4	4/4	0/4
5 Cycles 1 h each	0/4	3/4	0/4
	**Briotech**		
	**Pre-Contamination**	**Contamination**	**Post-Decontamination**
1 Cycle	0/4	4/4	0/4
10 Cycles	0/4	4/4	0/4
25 Cycles	0/4	4/4	0/4
100 Cycles	0/4	4/4	0/4
500 Cycles	0/4	4/4	0/4
1 Cycle 1 h	0/4	4/4	0/4
5 Cycles 1 h each	0/4	4/4	0/4
	**Virkon S**		
	**Pre-Contamination**	**Contamination**	**Post-Decontamination**
1 Cycle	1/4	2/4	4/4
10 Cycles	0/4	3/4	0/4
25 Cycles	0/4	4/4	1/4
100 Cycles	0/4	1/4	0/4
500 Cycles	0/4	3/4	0/4
1 Cycle 1 h	0/4	4/4	2/4
5 Cycles 1 h each	0/4	3/4	0/4
	**Wex-Cide**		
	**Pre-Contamination**	**Contamination**	**Post-Decontamination**
1 Cycle	0/4	4/4	0/4
10 Cycles	0/4	4/4	0/4
25 Cycles	0/4	4/4	0/4
100 Cycles	0/4	4/4	0/4
500 Cycles	0/4	3/4	2/4
1 Cycle 1 h	0/4	4/4	1/4
5 Cycles 1 h each	0/4	3/4	1/4

**Table 2:** Nonparametric statistical analyses of non-Gaussian swab data indicated that all chemicals except Virkon S effectively decontaminated knife surfaces. Statistically significant differences are shown in bold.

Decontaminant Condition	Kruskal–Wallis Statistic	Post Hoc Test	Dunn’s Multiple Comparison Test (Mean Rank Difference)
10% Bleach	**16.86; *p* < 0.0001**		
		Pre-Contamination vs. Contamination	**−11.50; *p* = 0.0004**
		Pre-Contamination vs. Post-Decontamination	−2.000; *p* > 0.9999
		Contamination vs. Post-Decontamination	**9.500; *p* = 0.0045**
40% Bleach	**15.43; *p* = 0.0004**		
		Pre-Contamination vs. Contamination	**−9.000; *p* = 0.0020**
		Pre-Contamination vs. Post-Decontamination	0.000; *p* > 0.9999
		Contamination vs. Post-Decontamination	**9.000; *p* = 0.0020**
Briotech	**18.97; *p* < 0.0001**		
		Pre-Contamination vs. Contamination	**−10.50; *p* = 0.0005**
		Pre-Contamination vs. Post-Decontamination	0; *p* > 0.999
		Contamination vs. Post-Decontamination	**10.50; *p* = 0.0005**
Virkon S	**13.15; *p* = 0.0004**		
		Pre-Contamination vs. Contamination	**−11.00; *p* = 0.0010**
		Pre-Contamination vs. Post-Decontamination	−4.000; *p* = 0.5781
		Contamination vs. Post-Decontamination	7.000; *p* = 0.0679
Wex-Cide	**14.99; *p* < 0.0001**		
		Pre-Contamination vs. Contamination	**−11.57; *p* = 0.0005**
		Pre-Contamination vs. Post-Decontamination	−3.429; *p* = 0.7925
		Contamination vs. Post-Decontamination	**8.143; *p* = 0.0240**

## Data Availability

The original contributions presented in this study are included in the article. Further inquiries can be directed to the corresponding author.

## Supplementary Materials

The following supporting information can be downloaded at https://www.mdpi.com/article/10.3390/pathogens15060645/s1. Figure S1. Nano-QuIC testing of deer venison used to contaminate knife surfaces; Figure S2. Recoverable CWD-prion seeding activity varies as a function of the decontaminant used for chemical aging; Figure S3. Screenshot of representative raw RT-QuIC data (48 h) from four Briotech-exposed knives; Figure S4. Screenshot of representative raw RT-QuIC data (48 h) from three 10% bleach-exposed knives. Prion-positive plate controls were enriched prion preparations generated from deer obex as described in [30].

## Abbreviations

The following abbreviations are used in this manuscript:

CWD	Chronic Wasting Disease
PrP^C^	Cellular Prion Protein
PrP^Sc^	Pathogenic Prion Protein
SEM	Scanning Electron Microscopy
EDS	Energy-Dispersive X-ray Analysis
RT-QuIC	Real-time Quaking Induced Conversion
RAF	Rate of Amyloid Formation
