# Supplementary material for: Impact of Chemical Aging on Venison Processing Knife Topography and Recoverable Chronic Wasting Disease Prion Seeding Activity
Source: Pathogens. 2026 Jun 17;15(6):645. doi: 10.3390/pathogens15060645 (PMC13304609; doi:10.3390/pathogens15060645)
Supplement: Supplementary file 1 [file pathogens-15-00645-s001.zip › pathogens-4358118-supplementary.pdf]

Supplemental Data

S1.

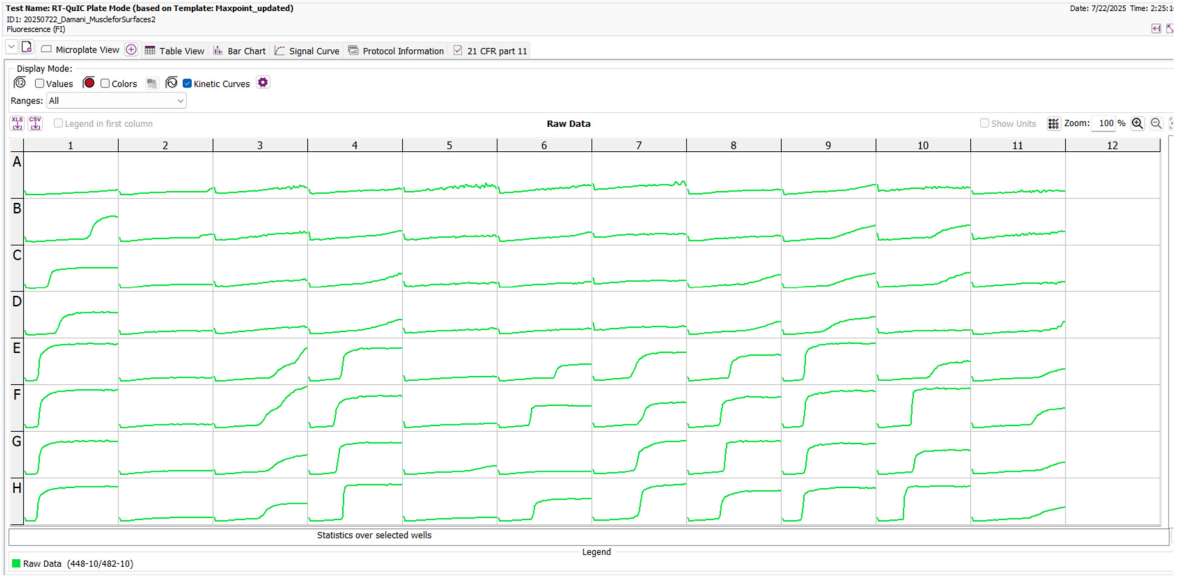

Supplementary Figure S1. Nano-QuIC testing of deer venison used to contaminate knife surfaces. Nine frozen venison samples were tested for seeding activity using Nano-QuIC. All samples were tested in quadruplicate. Rows A-D contain samples that are neat or 10^0. Rows E-H contain the same samples diluted 10-fold to 10^{-1}. Sample #59045 in column four was used to contaminate all knife surfaces. Columns one and two are positive and negative plate controls, respectively.

S2A.

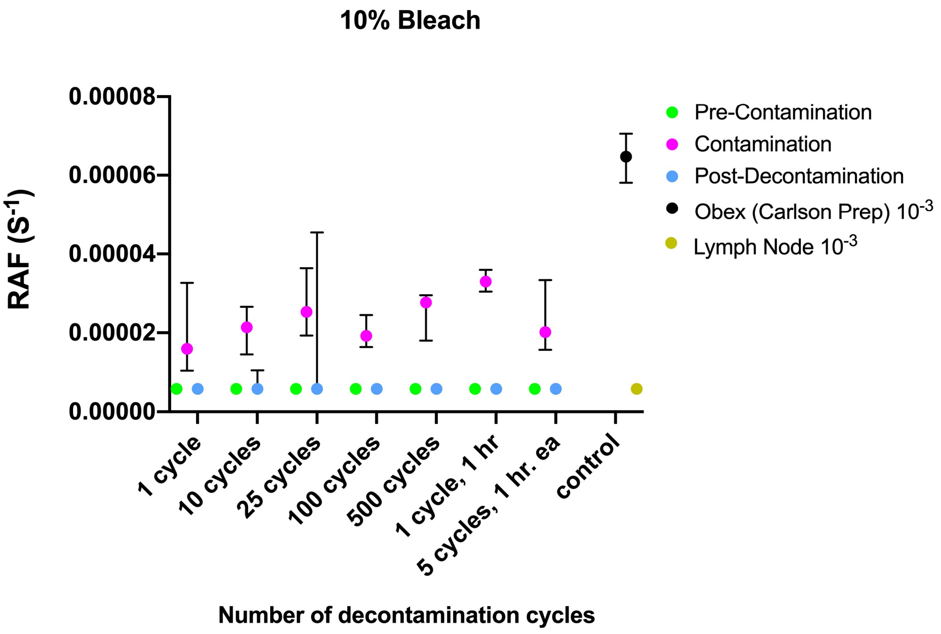
10% Bleach
0.00008
0.00006
0.00004
0.00002
0.00000
RAF (S-1)
Pre-Contamination
Contamination
Post-Decontamination
Obex (Carlson Prep) 10-3
Lymph Node 10-3
1 cycle
10 cycles
25 cycles
100 cycles
500 cycles
1 cycle, 1 hr
5 cycles, 1 hr. ea
control
Number of decontamination cycles

S2B.

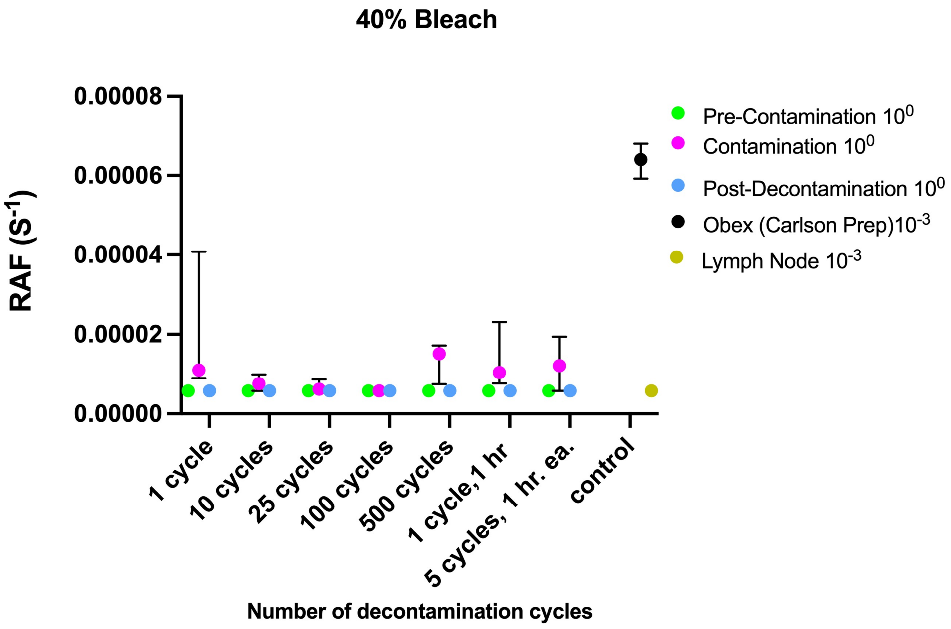
40% Bleach
0.00008
0.00006
0.00004
0.00002
0.00000
RAF (S-1)
Pre-Contamination 100
Contamination 100
Post-Decontamination 100
Obex (Carlson Prep)10-3
Lymph Node 10-3
1 cycle
10 cycles
25 cycles
100 cycles
500 cycles
1 cycle,1 hr
5 cycles, 1 hr. ea.
control
Number of decontamination cycles

S2C.

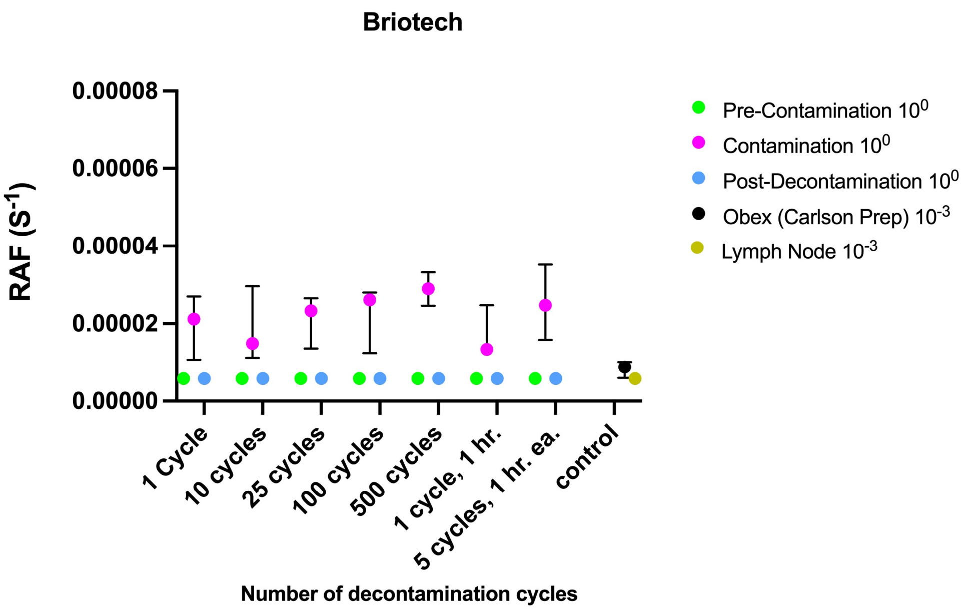
Briotech
0.00008
0.00006
0.00004
0.00002
0.00000
RAF (S-1)
1 Cycle
10 cycles
25 cycles
100 cycles
500 cycles
1 cycle, 1 hr.
5 cycles, 1 hr. ea.
control
Number of decontamination cycles
Pre-Contamination 10^0
Contamination 10^0
Post-Decontamination 10^0
Obex (Carlson Prep) 10^-3
Lymph Node 10^-3

S2D.

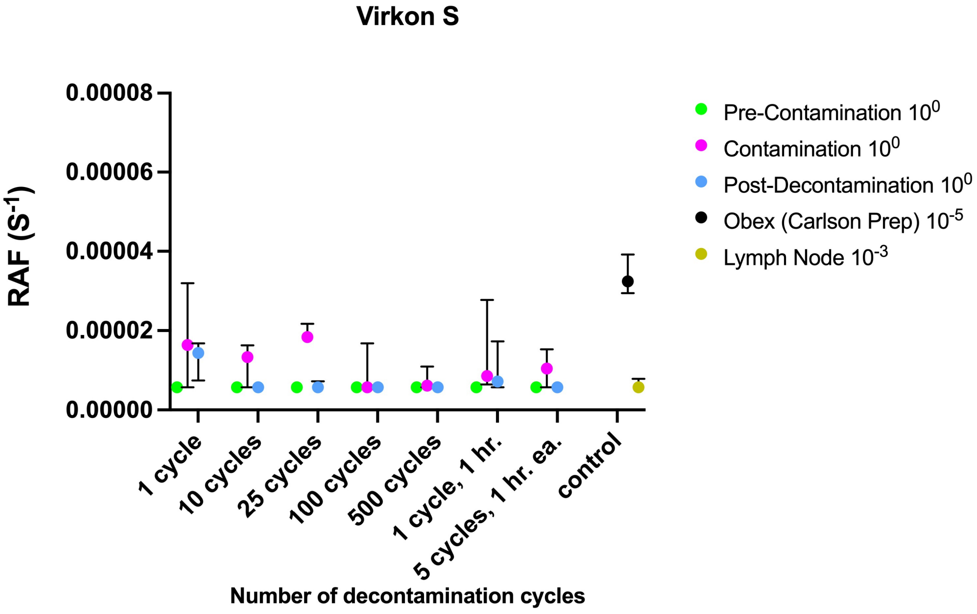
Virkon S
0.00008
0.00006
0.00004
0.00002
0.00000
RAF (S-1)
1 cycle
10 cycles
25 cycles
100 cycles
500 cycles
1 cycle, 1 hr.
5 cycles, 1 hr. ea.
control
Number of decontamination cycles
Pre-Contamination 10^0
Contamination 10^0
Post-Decontamination 10^0
Obex (Carlson Prep) 10^-5
Lymph Node 10^-3

S2E.

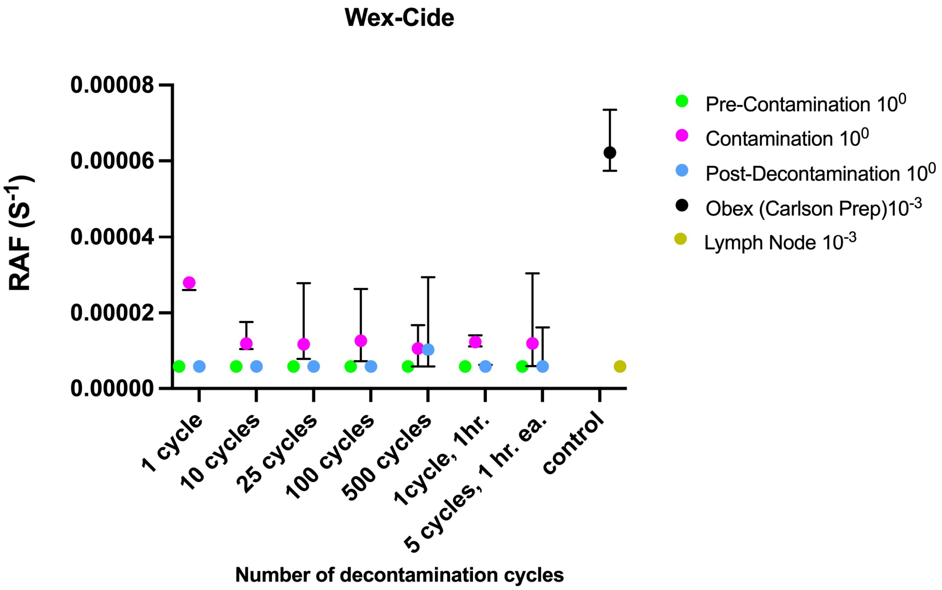

Supplemental Figure S2**. Recoverable CWD-prion seeding activity varies as a function of the decontaminant used for chemical aging.** Chemically aged knives were swabbed before contamination (green), after CWD prion contamination (magenta), and after 35 minutes of decontamination and a rinse with tap water (blue). Knives were previously chemically aged for the indicated number of cycles (x-axis) for five minutes each (ea) or 1-hour (hr) ea of prion decontamination. Each colored data point represents the median of 4 technical replicates from one swab. Vertical bars indicate the range. **(A)** 10% bleach was associated with 2 instances of decontamination failure (blue, 10 cycles, 25 cycles), **(B)** 40% bleach-treated knives evidenced decreased prion seeding (magenta, 10, 25, 100 cycles), **(C)** Briotech consistently decontaminated knives **(D)** Virkon S (blue, 1 cycle, one 1-hr cycle)**,** and **(E)** Wex-Cide (blue, 500 cycles, five 1-hr cycles) had two decontamination failures each**.** A Carlson preparation [30] of an obex from a CWD-positive deer (black) and a 10% homogenate of a deer lymph node (gold) were used as positive and negative plate controls, respectively.

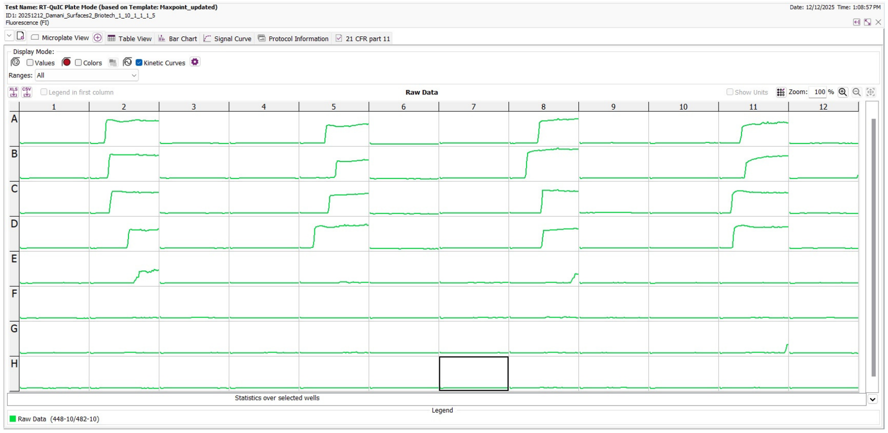

Supplemental Figure S3. Screenshot of representative raw RT-QuIC data (48 hrs.) from four Briotech exposed knives. The following conditions were tested in this RT-QuIC assay: one cycle (columns (cols) 1-3, 10 cycles (cols 4-6), one 1-hr cycle (cols 7-9), five 1-hr cycles (cols 9-12). Cols 1,4,7, and 10 contain data from swabs of knife blades prior to contamination. Cols 2,5,8, and 11 contain data from swabs of contaminated knife blades. Cols 3,6,9, and 12 are from swabs of post-decontaminated knife blades. Rows A-D are quadruplicate technical replicates from 10^0 samples. Rows E-H are quadruplicate technical replicates of the same sample diluted 10-fold to 10^{-1}. We consistently failed to observe seeding at this dilution, so this data was discarded.

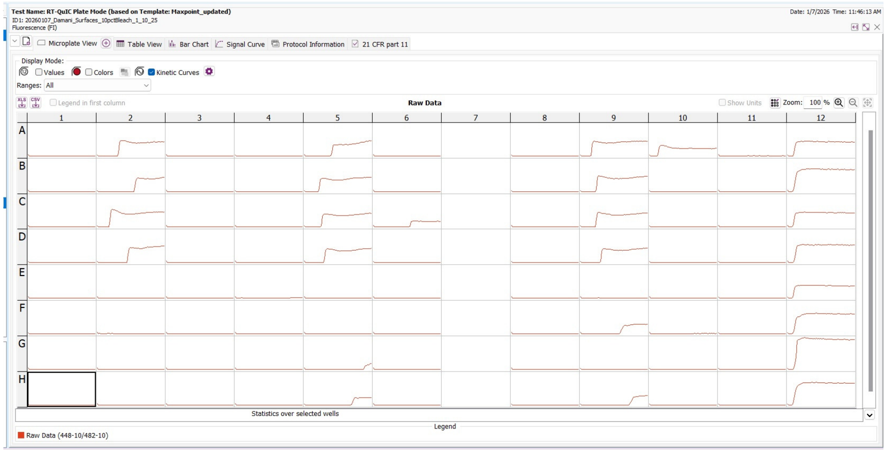

Supplemental Figure S4. Screenshot of representative raw RT-QuIC data (48 hrs.) from three 10% Bleach exposed knives. The following decontaminant conditions were tested in this RT-QuIC assay: one cycle (columns (cols) 1-3, 10 cycles (cols 4-6), 25 cycles (cols 8-10). Col 7 is unused and cols 11 and 12 are negative and positive plate controls, respectively. Cols 1,4, and 8 contain data from swabs collected prior to contamination. Cols 2,5, and 9 contain data from swabs of contaminated knives. Cols 3,6, and 10 were from swabs collected post-decontamination. Although seeding observed in col 6, row D and col 10, row A are quintessential examples of decontamination failures, they do not reach the threshold or prion positivity for those samples in the context of our semiquantitative analysis. Rows A-D are 4 technical replicates of each 10^0 sample. Rows E-H are 4 technical replicates of the same sample diluted 10-fold to 10^{-1}. We did not observe consistent seeding at this dilution, so this data was discarded.

Prion-positive plate controls were enriched prion preparations generated from deer obex as de-scribed in [30].
